# Supplementary material for: Factors associated with contrast-associated acute kidney injury in an emergency department: A cohort study in Lebanon
Source: PLoS One. 2025 Mar 13;20(3):e0316604. doi: 10.1371/journal.pone.0316604 (PMC11906086; doi:10.1371/journal.pone.0316604)
Supplement: Attachment — Submitted filename: Nephrotox_manuscript_PloS one.docx REVIEW COMMENTS.docx [file pone.0316604.s002.docx]

**Factors Associated with Contrast-Induced Acute Kidney Injury in a Tertiary Care Center Emergency Department: an Observational Cohort Study.**

Moustafa Al Hariri^1^, Sally Al Hassan^2^, Malak Khalifeh^2^, Hani Tamim^3,4^, Imad El Majzoub^5^, Tharwat El Zahran^2#^

^1^ College of Medicine, QU Health, Qatar University, Doha, Qatar

^2^ Department of Emergency Medicine, American University of Beirut Medical Center, Beirut, Lebanon

^3^ Department of Internal Medicine, American University of Beirut Medical Center, Beirut, Lebanon

^4^ College of Medicine, Alfaisal University, Riyadh, Saudi Arabia

^5^ Department of Emergency Medicine, Sheikh Shakhbout Medical City, Abu Dhabi, UAE

**Word count:** 2210

**# Corresponding author:**

*Tharwat El Zahran*, MD

Assistant professor of clinical specialty

Department of Emergency Medicine

American University of Beirut Medical Center

Beirut, Lebanon

P.O. Box 11-0236

Phone: 00961-1-350000 ext. 6602

<https://orcid.org/0000-0002-1182-2435>

[Te15@aub.edu.lb](mailto:Te15@aub.edu.lb)

**Abstract:**

**Background:** Contrast-induced acute kidney injury (CI-AKI) is a common problem in hospitals, particularly in low-middle-income countries (LMIC). Kidney function evaluation using serum creatinine levels before contrast administration leads to increased length of stay and delayed patient care. This study aimed to identify factors associated with CI-AKI in emergency department (ED) patients in an LMIC.

**Methods:** A retrospective cohort study was conducted at the largest tertiary care center's ED in Lebanon between November 2018 and December 2019. The study included ED patients who underwent computed-tomography (CT) with contrast. Bivariate and logistic regression analyses were performed to compare the characteristics of patients who developed AKI with those who did not. The Institutional Review Board (IRB) at the American University of Beirut (AUB) approved this study under protocol ID BIO-2020-0276, which was performed per the Declaration of Helsinki.

**Results:** The study included 1832 patients, of whom 10.4% (n=190) developed CI-AKI. Patients aged over 65 had a 1.6-fold higher risk of CI-AKI (aOR=1.55, 95%CI:1.09-2.2). High blood pressure (≥140 mmHg), high respiratory rate (≥ 22), and chronic kidney disease were significantly associated with CI-AKI. The use of loop diuretics (aOR=2.21, 95%CI:1.49-3.28), beta-lactams (aOR=4.11, 95%CI:2.63-6.42), and allopurinol (aOR=2.74, 95%CI:1.43-5.25) were significantly associated with CI-AKI.

**Conclusions:** Identifying factors associated with CI-AKI in an emergency setting, such as age, comorbidities, and home medications, can help identify patients at low risk of developing CI-AKI.

**Keywords:** Contrast-induced acute kidney injury, Emergency department, Nephrotoxicity, Kidney injury, Toxicity, Loop diuretics, and Beta-lactam.

**Background**

Contrast-induced acute kidney injury (CI-AKI) is the third most prevalent cause of hospital-acquired AKI and affects up to 30% of patients who receive iodinated contrast media. The total impairment to patients' health and public expenses related to CI-AKI is significant, with more than 30 million doses of iodinated contrast medium administered annually (1).

The pathogenesis behind CI-AKI is contrast-induced vasoconstriction which decreases the blood supply to the renal medulla. Nevertheless, comorbidities such as diabetes mellitus, congestive heart failure, acute hypotension (requiring pressors or intra-aortic balloon pump), ST-elevation myocardial infarction, and volume depletion in patients at risk for CI-AKI worsen the basic etiology of the injury. Additionally, patients with chronic kidney disease are more prone to develop CI-AKI since they have a decreased number of nephrons and their compensatory mechanisms to maintain filtration performance are weakened. Additional risk factors include procedural difficulties, including the amount of contrast used and the type of contrast used (1). Risk factor scoring of both baseline comorbidities and procedural factors has been used as predictors for CI-AKI, the need for renal replacement therapy, and long-term mortality (2).

One way that has been proposed to avoid CI-AKI is volume expansion via intravenous fluid infusion to increase urine flow. In hospitalized patients, this can be achieved by starting the intravenous infusion 6-12 hours before administering the contrast material and counting for at least 4 hours thereafter. However, in the emergency setting, this is challenging as patients require immediate imaging and prompt diagnosis of a life-threatening condition (1).

Measuring serum creatinine is used to assess kidney function in patients before contrast administration. Nevertheless, measuring serum creatinine on every ED patient adds a financial burden on the patient, increases the wait time for prompt diagnosis, increases the length of ED stay, and delays care (3). Most patients presenting to the ED have normal creatinine levels, and this subset group can be identified using a risk assessment questionnaire (4).

**Methods:**

**Aim**

This study aims to assess the factors associated with the development of CI-AKI in patients who received computed tomography (CT) scans with contrast during their presentation to an academic Emergency Department in a tertiary care center.

**Study Design and Setting:**

This study was an IRB-approved, retrospective, observational cohort study conducted between November 2018 and December 2019. This study was conducted at the Department of Emergency Medicine (DEM) of the American University of Beirut Medical Center (AUBMC), the largest academic and tertiary care center in a Lebanon, a low-middle-income country. The Institutional Review Board (IRB) at the American University of Beirut (AUB) approved this study under protocol ID BIO-2020-0276 in July 2020 and the study was performed in accordance with the Declaration of Helsinki. The medical record team at AUBMC provided the authors with the patient list and data in August 2021. Although the exported data contained the identifiers of participants (medical record number, and date and time of ED arrival) we did not have to access the medical records of the patients since the exported data by the medical record team contained all needed study variables. No participant identifiers are included in this manuscript. This study conforms to the Strengthening the Reporting of Observational Studies in Epidemiology (STROBE) guidelines.

**Population:**

Patients presenting to the DEM who received Computed Tomography (CT) with contrast (CI-CT) as a clinical workup were identified by the Electronic Health Record (EPIC) team in August 2021. Study participants received 2ml/Kg contrast solution on average. We excluded the records of the patients that lacked baseline creatinine level and lacked creatinine level assessment within 48 hours post CI-CT. This study did not involve patients in its design for its retrospective observational nature. However, we plan to share the study’s findings with healthcare professionals through different routes.

**Outcome Measures:**

Patients’ details were prepared as reports by the EPIC team. Data management and merging were performed to collate all participants’ variables longitudinally. Participants’ variables included demographics, past medical and surgical history, home medication, vitals, ED management (medications and IV administration), home medications, and laboratory workup. We included the records of the patients up to two days before the contrast administration and up to 7 days post contrast administration to collect the data on the IV-fluid administration and creatinine and/or eGFR laboratory values to be able to assess AKI. Cutoffs for Systolic blood pressure (≥140 mmHg) (5), Heart rate (≥100 bps) (6), and respiratory rate (≥22 bps) (7) were implemented in the analysis according to the values defined in our institution and previous reports in the literature.

**AKI definitions**

This study used the Kidney Disease Improving Global Outcomes (KDIGO) definition for AKI, which defines AKI as an increase in creatinine level of 0.3 mg/dl (26 mmol/l) within 48 hours or a 50% increase over the baseline level within 7 days (8). The records of the identified participants who did not have at least one creatinine measurement within 48 hours of CI-CT were excluded from the study.

**Sample size:**

We included the records of all the participants who fulfilled the inclusion criteria of the study, which were 1832 participants.

**Data Analysis:**

Data analysis was performed using SPSS (IBM SPSS 25.0). Categorical variables were presented using frequencies and percentages, while continuous variables were presented using mean and standard deviation (mean ± SD). To observe the associations between the different categorical variables and our outcome, Pearson’s Chi-square and Fisher’s exact tests, where applicable, were used for categorical variables and Student’s T-test for continuous ones. To account for multiple comparisons, q values (FDR) were calculated and reported. Logistic regression was then done to adjust confounding variables and test the association between contrast-induced AKI and other variables. Variables that showed a significant confidence interval of the unadjusted OR and clinically relevant were selected to build the logistic regression model. Significance was interpreted at α ≤0.05.

**Results:**

**Baseline Characteristics:**

In this study, a total of 1832 patients were included, among which 10.4% developed contrast-induced AKI (n=190). The median age of the participants was 60 years old (IQR: 42.39-74), and 51.7% were females (n=948). Only 43% reported being smokers (n=787), and 25.8% reported being alcohol consumers (n=189). Cardiovascular diseases were the most prevalent medical history (n=538, 29.4%) followed by cancer (n=651, 35.5%), hypertension (n=333, 18.2%), diabetes (n=81, 4.4%) and liver diseases (n=132, 7.2%) (Table 1).

**Table 1: Association of Baseline Characteristics and AKI**

|  | **options** | **Total**  **N=1832** | **No AKI**  **N=1642 (%)** | **AKI**  **N=190 (%)** | **p-value** | **q-Value** | **OR** | **95%CI** |
| --- | --- | --- | --- | --- | --- | --- | --- | --- |
| **Age** | **< 60 years** | 915 (49.9%) | 854 (52%) | 61 (32.1%) | < 0.001 | <0.001 | Ref |  |
|  | **≥ 60 years** | 917 (50.1%) | 788 (48%) | 129 (67.9%) |  |  | 2.29 | 1.67-3.16 |
| **Gender** | **Female** | 948 (51.7%) | 858 (52.3%) | 90 (47.4%) | 0.202 | 0.358 | Ref |  |
|  | **Male** | 884 (48.3%) | 784 (47.7%) | 100 (52.6%) |  |  | 1.22 | 0.9-1.64 |
| **Alcohol** |  | 189 (25.8%) | 172 (25.8%) | 17 (25.8%) | 0.512 | 0.713 | 1.00 | 0.56-1.78 |
| **Smoking** |  | 787 (43%) | 715 (43.5%) | 72 (37.9%) | 0.136 | 0.280 | 0.79 | 0.58-1.08 |
| **Comorbidities:** | **Cardiovascular diseases** | 538 (29.4%) | 455 (27.7%) | 83 (43.7%) | < 0.001 | <0.001 | 2.02 | 1.49-2.75 |
|  | **Hypertension** | 333 (18.2%) | 288 (17.5%) | 45 (23.7%) | 0.038 | 0.086 | 1.46 | 1.02-2.09 |
|  | **Diabetes** | 181 (9.9%) | 154 (9.4%) | 27 (14.2%) | 0.035 | 0.084 | 1.60 | 1.03-2.48 |
|  | **Cancer** | 651 (35.5%) | 575 (35%) | 76 (40%) | 0.174 | 0.340 | 1.24 | 0.91-1.68 |
|  | **Rheumatological disease** | 38 (2.1%) | 37 (2.3%) | 1 (0.5%) | 0.173 | 1.000 | 0.23 | 0.03-1.68 |
|  | **Chronic Kidney Disease** | 81 (4.4%) | 53 (3.2%) | 28 (14.7%) | < 0.001 | <0.001 | 5.18 | 3.19-8.42 |
|  | **Organ Transplant** | 67 (3.7%) | 58 (3.5%) | 9 (4.7%) | 0.402 | 0.654 | 1.36 | 0.66-2.79 |
|  | **Liver disease** | 132 (7.2%) | 110 (6.7%) | 22 (11.6%) | 0.014 | 0.041 | 1.82 | 1.12-2.96 |

Data are presented as numbers with percentages.

The p-value for the difference between AKI vs. no AKI is calculated by chi-square, Fisher´s exact test, or T-test where appropriate.

The q-value is the False Discovery Rate (FDR) significance accounting for multiple comparisons.

Additionally, most participants had normal baseline eGFR (≥65 ml/min) (n= 1540, 87.6%). A minority had a high qSOFA score of either 1 (20.1%) or 2 (2.3%). Table 2 shows different concomitant nephrotoxic medications taken by the patients. About 22.9% were on aspirin (n=419),12.2% were on loop diuretics (n=224), 12.2% on vancomycin (n=224) followed by thiazide diuretic (n=140, 7.6%), beta-lactam antibiotics (n=134, 7.3%), allopurinol (n=63, 3.4%), and NSAIDs (n=47, 2.6%) (Table 2).

**Table 2: Association of Clinical Characteristics and AKI**

|  | options | Total  N=1832 | No AKI  N=1642 (%) | AKI  N=190 (%) | p-value | q-value | OR | 95%CI |
| --- | --- | --- | --- | --- | --- | --- | --- | --- |
| qSOFA | **0** | 1419 (77.6%) | 1305 (79.6%) | 114 (60%) | < 0.001 | <0.001 | Ref |  |
|  | **1** | 368 (20.1%) | 300 (18.3%) | 68 (35.8%) |  |  | 2.60 | 1.87-3.59 |
|  | **2** | 42 (2.3%) | 34 (2.1%) | 8 (4.2%) |  |  | 2.69 | 1.22-5.96 |
| Systolic Blood Pressure (SBP) | **< 140** | 1218 (66.6%) | 1107 (67.5%) | 111 (58.4%) | 0.012 | 0.038 | Ref |  |
|  | **≥ 140** | 611 (33.4%) | 532 (32.5%) | 79 (41.6%) |  |  | 1.48 | 1.09-2.01 |
| Heart Rate (HR) | **< 100** | 1111 (60.7%) | 994 (60.6%) | 117 (61.6%) | 0.803 | 1.000 | Ref |  |
|  | **≥ 100** | 718 (39.3%) | 645 (39.4%) | 73 (38.4%) |  |  | 0.96 | 0.71-1.31 |
| Respiratory Rate (RR) | **< 22** | 1531 (83.7%) | 1404 (85.7%) | 127 (66.8%) | < 0.001 | <0.001 | Ref |  |
|  | **≥ 22** | 298 (16.3%) | 235 (14.3%) | 63 (33.2%) |  |  | 2.96 | 2.13-4.13 |
| Received IV Hydration prior to CT | | 1800 (98.3%) | 1613 (98.2%) | 187 (98.4%) | 1.000 | 1.000 | 0.85 | 0.34-3.71 |
| Performed Angio CT | | 436 (23.8%) | 378 (23.0%) | 58 (30.5%) | 0.022 | 0.056 | 1.47 | 1.06-2.04 |
| Concomitant Nephrotoxic Medications: | **Antiviral** | 41 (2.2%) | 34 (2.1%) | 7 (3.7%) | 0.187 | 0.348 | 1.81 | 0.79-4.14 |
|  | **Antiretroviral** | 1 (0.1%) | 1 (0.1%) | 0 (0%) | 1.000 | 1.000 |  |  |
|  | **Loop Diuretics** | 224 (12.2%) | 163 (9.9%) | 61 (32.1%) | < 0.001 | <0.001 | 4.29 | 3.04-6.06 |
|  | **Thiazide Diuretics** | 140 (7.6%) | 123 (7.5%) | 17 (8.9%) | 0.474 | 0.712 | 1.21 | 0.71-2.06 |
|  | **ACEI** | 55 (3%) | 51 (3.1%) | 4 (2.1%) | 0.444 | 0.693 | 0.67 | 0.24-1.88 |
|  | **Chemotherapy*** | 12 (0.7%) | 11 (0.7%) | 1 (0.5%) | 1.000 | 1.000 | 0.79 | 0.10-6.11 |
|  | **Immunotherapy**** | 7 (0.4%) | 7 (0.4%) | 0 (0%) | 1.000 | 1.000 |  |  |
|  | **NSAID** | 47 (2.6%) | 43 (2.6%) | 4 (2.1%) | 1.000 | 1.000 | 0.8 | 0.28-2.25 |
|  | **Aminoglycoside** | 66 (3.6%) | 53 (3.2%) | 13 (6.8%) | 0.016 | 0.044 | 2.20 | 1.18-4.12 |
|  | **Beta Lactam** | 134 (7.3%) | 84 (5.1%) | 50 (26.3%) | < 0.001 | <0.001 | 6.62 | 4.48-9.79 |
|  | **Sulfonamides** | 18 (1.0%) | 14 (0.9%) | 4 (2.1%) | 0.108 | 0.234 | 1.07 | 0.58-2.00 |
|  | **Antiepileptic** | 26 (1.4%) | 25 (1.5%) | 1 (0.5%) | 0.511 | 0.731 | 0.34 | 0.05-2.54 |
|  | **Allopurinol** | 63 (3.4%) | 47 (2.9%) | 16 (8.4%) | < 0.001 | <0.001 | 3.12 | 1.73-5.62 |
|  | **Dapsone** | 2 (0.1%) | 2 (0.1%) | 0 (0%) | 1.000 | 1.000 |  |  |
|  | **Amitryptilline** | 16 (0.9%) | 14 (0.9%) | 2 (1.1%) | 0.678 | 0.912 | 1.24 | 0.28-5.49 |
|  | **Lanzoprazole** | 9 (0.5%) | 8 (0.5%) | 1 (0.5%) | 1.000 | 1.000 | 1.08 | 0.13-8.69 |
|  | **Rifampicin** | 3 (0.2%) | 2 (0.1%) | 1 (0.5%) | 0.28 | 0.475 | 4.34 | 0.39-48.07 |
|  | **Tetracycline** | 2 (0.1%) | 2 (0.1%) | 0 (0%) | 1.000 | 1.000 |  |  |
|  | **Lithium** | 6 (0.3%) | 6 (0.4%) | 0 (0%) | 1.000 | 1.000 |  |  |
|  | **Aspirin** | 419 (22.9%) | 361 (22%) | 58 (30.5%) | 0.008 | 0.028 | 1.56 | 1.12-2.17 |
|  | **Vancomycin** | 224 (12.2%) | 177 (10.8%) | 47 (24.7%) | < 0.001 | < 0.001 | 2.72 | 1.89-3.92 |
|  | **Colistin** | 36 (2%) | 22 (1.3%) | 14 (7.4%) | < 0.001 | < 0.001 | 5.86 | 2.94-11.65 |

Data are presented as numbers with percentages.

* Chemotherapy includes Cisplatin, Carboplatin, Cyclophosphamide, Ifosfamide, and Methotrexate. ** Immunotherapies include Sirolimus, Tacrolimus, and Cyclosporine.

Abbreviations: ACEI: Angiotensin-converting enzyme Inhibitor and NSAID: Non-steroidal anti-inflammatory drugs.

The p-value for the difference between AKI vs. no AKI is calculated by chi-square, Fisher´s exact test, or T-test where appropriate.

The q-value is the False Discovery Rate (FDR) significance accounting for multiple comparisons.

**Characteristics of patients with contrast-induced AKI:**

Patients with AKI were older than 65 (58.9% vs. 37.4%, p < 0.001). However, there was no significant difference in gender, smoking status, and alcohol consumption status with contrast-induced AKI. As for the types of comorbidities associated with CI-AKI, cardiovascular diseases (43.7% vs. 27.7%, p<0.001) showed a significant association. Other comorbidities showed significant association with CI-AKI, including hypertension (23.7% vs. 17.5%, p=0.038), diabetes (14.2% vs. 9.4%, p=0.035), chronic kidney disease (14.7% vs. 3.2%, p<0.001), and liver diseases (11.6% vs. 6.7%, p=0.014) (Table 1).

As for vital signs, systolic blood pressure and respiratory rate were significantly associated with CI-AKI. Patients with high blood pressure (≥140 mmHg) and high respiratory rate (≥ 22) were more associated with CI-AKI.

As summarized in table 2, a significantly higher portion of the CI-AKI patients reported to be on loop diuretics (32.1% vs. 9.9%, p < 0.001), aminoglycosides (6.8% vs. 3.2%, p=0.011), beta-lactams (26.3% vs. 5.1%, p < 0.001), allopurinol (8.4% vs. 2.9%, p < 0.001), lithium (30.5% vs. 22%, p=0.008), aspirin (24.7% vs. 10.8%, p < 0.001), and colistin (7.4% vs. 1.3%, p<0.001). Patients with CI-AKI had significantly higher qSOFA score either 1 (35.8% vs. 18.3%, p<0.001) or 2 (3.7% vs. 2.3%, p<0.001). The percentages of patients with CI-AKI were higher in patients with lower baseline eGFR, as shown in Figure 1 (p<0.001).

**Factors associated with contrast-induced AKI:**

After adjusting for confounding variables using logistic regression, patients greater than 65 years old were about 1.6 times more associated with CI-AKI (aOR=1.55, 95%CI: 1.09-2.2). Patients with high blood pressure (≥140 mmHg) and high respiratory rate (≥ 22) were more associated with CI-AKI (aOR=1.34, 95%CI: 0.96-1.89, and aOR=2.03, 95%CI: 1.40-2.94, respectively). Patients with chronic kidney disease were about 4 times more associated with contrast-induced AKI (aOR=3.75, 95%CI: 2.18-6.45). Moreover, patients with CI-AKI were more on loop diuretics (aOR=2.21, 95%CI: 1.49-3.28), beta-lactams (aOR=4.11, 95%CI: 2.63-6.42), and allopurinol (aOR=2.74, 95%CI: 1.43-5.25) (Table 3).

**Table 3: Multivariable Logistic Regression: factors associated with AKI.**

|  | **aOR** | **95% C.I.** | | **P value** |
| --- | --- | --- | --- | --- |
| **Age (≥ 60 years)** | 1.547 | 1.091 | 2.195 | 0.014 |
| **SBP (≥ 140)** | 1.344 | 0.957 | 1.886 | 0.088 |
| **RR (≥ 22)** | 2.029 | 1.401 | 2.940 | 0.000 |
| **Chronic Kidney Disease** | 3.746 | 2.176 | 6.450 | 0.000 |
| **Loop Diuretic** | 2.206 | 1.486 | 3.276 | 0.000 |
| **Beta-Lactams** | 4.106 | 2.626 | 6.420 | 0.000 |
| **Allopurinol** | 2.739 | 1.430 | 5.245 | 0.002 |
| **Vancomycin** | 1.463 | 0.950 | 2.253 | 0.084 |

Variables entered in the model: Age (reference < 60 years), qSOFA (reference: 0), Diabetes, Hypertension, Cardiovascular diseases, Chronic Kidney Disease, Liver disease, Loop Diuretic, Aminoglycoside, Beta-Lactam, Allopurinol, Aspirin, Vancomycin, Colistin, Angiogram CT (reference: No), SBP (reference: <140), and RR (reference: < 22).

Omnibus test <0.001, R2=0.188, Hosmer=0.283; Variables entered < 0.05 in bivariate analysis

Abbreviations: SBP: Systolic Blood Pressure, RR: Respiratory Rate.

**Discussion:**

More than 35 definitions exist to define AKI. Still, in this study, we have adopted the KDIGO criteria, which define AKI either by an increase in serum creatinine by ≥0.3 mg/dl within 48 hours (9). We aimed to investigate the factors that are associated with contrast-induced nephrotoxicity (CIN) in an emergency setting at a tertiary care center.

Our study showed that age, hypertension, high respiratory rate, chronic kidney disease, use of loop diuretics, beta-lactam, and allopurinol were significantly and positively associated with the development of CIN. The association between age and CIN is reported in the literature. Recently, Lie et al. showed in their cohort study on 643 patients that age above 70 years is associated with CIN (10). Additional similar observations were reported by Briguori et al. and Donnarumma et al. (11, 12). Moreover, hypertension is also reported to be a risk factor and is associated with CIN (10, 13, 14). Similarly in our results, there was an association between hypertension and CIN.

As for the high respiratory rate, very few reports in the literature assessed the association between RR and CIN. However, Davenport et al. reported in a retrospective study conducted on more than 20,000 patients that sepsis was associated with CIN (15). Moreover, McCullough previously reviewed the association between sepsis and CIN (16). Since a high respiratory rate (RR>22) goes into the calculation of the qSOFA score and indicates sepsis, our findings align with the literature (17).

Chronic kidney disease is another risk factor for CIN development. Shinoura et al., in a cohort study on 1475 ED patients, reported that a high serum creatinine level (above 1.5 mg/dl) before CT with contrast is associated with CIN (18). Additionally, multiple reports showed that chronic kidney disease is among the risk factors for CIN development (19-21).

Previous studies have reported using angiotensin-converting enzyme inhibitors (ACEIs) and loop diuretics as risk factors for CIN. The proposed mechanism is the reduction of adequate intravascular volume, which leads to prerenal perfusion reduction and intrarenal vasoconstriction (22). Our findings showed that patients on loop antidiuretics were 2 times more likely to develop CIN. However, when it comes to ACEIs, we have found no significant difference in CIN between those who were on ACEIs and those who weren’t.

In previous literature, there are contradicting findings related to the effect of Allopurinol with CIN. For instance, Rey et al. conducted a post-marketing study to assess the association between Allopurinol and AKI cases. The findings of this study confirm that Allopurinol increases the odds of developing AKI (23). Our results showed a close association between Allopurinol and AKI with Rey et al. report. On the other hand, many reports in the literature support the idea that Allopurinol decreases the risk of CI-AKI. Ma et al. conducted a meta-analysis on 176 randomized clinical trials and showed that pretreatment with Allopurinol significantly reduced the incidence of CI-AKI (relative risk (RR): 0.37, p = 0.01) (24). This contrasted with our findings that have shown that patients on Allopurinol were 2 times more likely to develop CI-AKI.

We found that using Beta-lactams has significantly increased the risk for CI-AKI. Patients taking Beta-lactams were 4 times more likely to develop CI-AKI. This is no surprise, as the use of nephrotoxic drugs was shown to be a risk factor for CI-AKI in many published studies (25, 26). Moreover, publications emphasizing the nephrotoxicity of Beta lactams antibiotics, especially on proximal tubular cells, are many (27-32).

Additionally, a cross-sectional study in China found that the concomitant use of vancomycin with contrast material was significantly associated with the development of AKI (33). This is in line with our results, where we have found that Vancomycin use increases the risk of CI-AKI by 1.4 times. These results aren’t surprising as Vancomycin has oxidative effects.

**Limitations:**

As for our study’s limitations, it is a single-center cohort study, which relied on the patients’ data in their medical charts. Although we only included the patients who presented to one care center, our Emergency Department is one of the country’s busiest, allowing our results to be extended to other care centers. For some patients, the retrospective nature of the study limited the authors’ ability to have more than one creatinine measurement to check for the AKI definition and include them in the analysis of the study.

On the other hand, the selection bias in our study was reduced by the fact that around 17% of the study participants exhibited an abnormal (above and lower the normal range) baseline creatinine level. Among these 306 participants, only 42 fulfilled the KODIGO definition of AKI. On another note, this study’s results pinpoint the need to design and conduct clinical trials to confirm the characteristics of the patients that are protected against CI-AKI and could benefit from rapid CI-CT in acute settings.

**Conclusion:**

In an emergency setting, assessing the factors associated with CI-AKI, such as age, high respiratory rate, chronic kidney disease, use of Beta-lactams, loop diuretics, and/or Allopurinol could help reduce the need to order serum creatinine lab assessment, expedite the imaging workup, and promptly diagnose life-threatening conditions. On another note, the results of this study indicate the need to develop clinical trial(s) to assess the safety of administering contrast substance(s) to patients without waiting for the creatinine level of the patients.

**List of abbreviations:**

AKI: Acute Kidney Injury

aOR: Adjusted Odds Ratio

AUB: American University Of Beirut

AUBMC: American University Of Beirut Medical Center

ACEI: Angiotensin-Converting Enzyme Inhibitors

CT: Computed-Tomography

CI-AKI: Contrast-Induced Acute Kidney Injury

CIN: Contrast-Induced Nephrotoxicity

DEM: Department Of Emergency Medicine

ED: Emergency Department

eGFR: Estimated Glomerular Filtration Rate

IRB: Institutional Review Board

IQR: Interquartile Range

KDIGO: Kidney Disease Improving Global Outcomes

LMIC: Low-Middle-Income Countries

NSAID: Non-Steroidal Anti-Inflammatory Drug

RR: Respiratory Rate

STROBE: Strengthening the Reporting of Observational Studies in Epidemiology

SBP: Systolic Blood Pressure

**References:**

1.	Fähling M, Seeliger E, Patzak A, Persson PB. Understanding and preventing contrast-induced acute kidney injury. Nat Rev Nephrol. 2017;13(3):169-80.

2.	Solomon R, Dauerman HL. Acute Kidney Injury: Culprits, Cures, and Consequences. Circ Cardiovasc Interv. 2017;10(1):e004742.

3.	Thomsen HS, Morcos SK. In which patients should serum creatinine be measured before iodinated contrast medium administration? Eur Radiol. 2005;15(4):749-54.

4.	Thomsen HS, Morcos SK. In which patients should serum creatinine be measured before iodinated contrast medium administration? European radiology. 2005;15(4):749-54.

5.	Basile JN. Systolic blood pressure. BMJ. 2002;325(7370):917-8.

6.	Avram R, Tison GH, Aschbacher K, Kuhar P, Vittinghoff E, Butzner M, et al. Real-world heart rate norms in the Health eHeart study. NPJ Digit Med. 2019;2:58-.

7.	Okawa R, Yokono T, Koyama Y, Uchiyama M, Oono N. Clinical Sign-Based Rapid Response Team Call Criteria for Identifying Patients Requiring Intensive Care Management in Japan. Medicina (Kaunas). 2021;57(11):1194.

8.	Thomas ME, Blaine C, Dawnay A, Devonald MA, Ftouh S, Laing C, et al. The definition of acute kidney injury and its use in practice. Kidney Int. 2015;87(1):62-73.

9.	Singbartl K, Kellum JA. AKI in the ICU: definition, epidemiology, risk stratification, and outcomes. Kidney international. 2012;81(9):819-25.

10.	Lei L, Xue Y, Guo Z, Liu B, He Y, Liu J, et al. Nomogram for contrast-induced acute kidney injury in patients with chronic kidney disease undergoing coronary angiography in China: a cohort study. BMJ open. 2020;10(5):e037256.

11.	Briguori C, Donnarumma E, Quintavalle C, Fiore D, Condorelli G. Contrast-induced acute kidney injury: potential new strategies. Curr Opin Nephrol Hypertens. 2015;24(2):145-53.

12.	Briguori C, Condorelli G. Hydration in contrast-induced acute kidney injury. Lancet. 2014;383(9931):1786-8.

13.	Pflueger A, Abramowitz D, Calvin AD. Role of oxidative stress in contrast-induced acute kidney injury in diabetes mellitus. Medical science monitor: international medical journal of experimental and clinical research. 2009;15(6):RA125-36.

14.	Kusirisin P, Chattipakorn SC, Chattipakorn N. Contrast-induced nephropathy and oxidative stress: mechanistic insights for better interventional approaches. J Transl Med. 2020;18(1):400.

15.	Davenport MS, Khalatbari S, Dillman JR, Cohan RH, Caoili EM, Ellis JH. Contrast material-induced nephrotoxicity and intravenous low-osmolality iodinated contrast material. Radiology. 2013;267(1):94-105.

16.	McCullough PA, Soman SS. Contrast-induced nephropathy. Crit Care Clin. 2005;21(2):261-80.

17.	Shahsavarinia K, Moharramzadeh P, Arvanagi RJ, Mahmoodpoor A. qSOFA score for prediction of sepsis outcome in emergency department. Pak J Med Sci. 2020;36(4):668-72.

18.	Shinoura S, Tokushige A, Chinen K, Mori H, Kato S, Ueda S. Association between contrast-induced nephrotoxicity and contrast enhanced computed tomography followed by endoscopic retrograde cholangiopancreatography. Eur J Radiol. 2020;129:109074.

19.	Krasinski Z, Krasińska B, Olszewska M, Pawlaczyk K. Acute Renal Failure/Acute Kidney Injury (AKI) Associated with Endovascular Procedures. Diagnostics (Basel, Switzerland). 2020;10(5).

20.	Caiazza A, Russo L, Sabbatini M, Russo D. Hemodynamic and tubular changes induced by contrast media. BioMed research international. 2014;2014:578974.

21.	Santos RO, Malvar B, Silva R, Ramalho V, Pessegueiro P, Amoedo M, et al. [Contrast-induced nephropathy]. Acta Med Port. 2011;24(5):809-20.

22.	Hossain MA, Costanzo E, Cosentino J, Patel C, Qaisar H, Singh V, et al. Contrast-induced nephropathy: Pathophysiology, risk factors, and prevention. Saudi Journal of Kidney Diseases and Transplantation. 2018;29(1):1.

23.	Rey A, Batteux B, Laville SM, Marienne J, Masmoudi K, Gras-Champel V, et al. Acute kidney injury associated with febuxostat and allopurinol: a post-marketing study. Arthritis Res Ther. 2019;21(1):229.

24.	Ma G, Wang G, Xiao D, Teng W, Hui X, Ma G. Meta-analysis on allopurinol preventive intervention on contrast-induced acute kidney injury with random controlled trials: PRISMA. Medicine. 2019;98(25).

25.	Seeliger E, Sendeski M, Rihal CS, Persson PB. Contrast-induced kidney injury: mechanisms, risk factors, and prevention. European Heart Journal. 2012;33(16):2007-15.

26.	Stacul F, van der Molen AJ, Reimer P, Webb JAW, Thomsen HS, Morcos SK, et al. Contrast induced nephropathy: updated ESUR Contrast Media Safety Committee guidelines. European radiology. 2011;21(12):2527-41.

27.	Kaloyanides GJ. Antibiotic-related nephrotoxicity. Nephrol Dial Transplant. 1994;9 Suppl 4:130-4.

28.	Khalili H, Bairami S, Kargar M. Antibiotics induced acute kidney injury: incidence, risk factors, onset time and outcome. Acta Medica Iranica. 2013:871-8.

29.	Morales-Alvarez MC. Nephrotoxicity of antimicrobials and antibiotics. Advances in Chronic Kidney Disease. 2020;27(1):31-7.

30.	Tune B. The nephrotoxicity of-lactam antibiotics. Toxicology of the Kidney. 1993:257-81.

31.	Tune B. Renal tubular transport and nephrotoxicity of beta lactam antibiotics: structure-activity relationships. Mineral and electrolyte metabolism. 1994;20(4):221-31.

32.	Tune BM. Nephrotoxicity of beta-lactam antibiotics: mechanisms and strategies for prevention. Pediatric Nephrology. 1997;11(6):768-72.

33.	Pan K, Ma L, Xiang Q, Li X, Li H, Zhou Y, et al. Vancomycin-associated acute kidney injury: a cross-sectional study from a single center in China. PloS one. 2017;12(4):e0175688.

**Ethical Statement:** The Institutional Review Board (IRB) at the American University of Beirut (AUB) approved this study under protocol ID BIO-2020-0276 and the study was performed in accordance with the Declaration of Helsinki. The IRB approved waiving the consent process for the participants for the impracticability of the process since it is a retrospective study, and the study investigators are no longer following-up the participants of the study.

**Consent for publication:** Not applicable.

**Data Availability:** The datasets generated during and/or analysed during the current study are available from the corresponding author on reasonable request.

**Competing Interests:** The authors declare they have no financial interests.

**Source of Funding:** No funding was received to assist with the preparation of this manuscript.

**Author contribution:** Conceptualization: Moustafa Al Hariri, Imad El Majzoub, and Tharwat El Zahran; Methodology: Moustafa Al Hariri, Imad El Majzoub, Hani Tamim, and Tharwat El Zahran; Formal analysis and investigation: Moustafa Al Hariri, Malak Khalifeh, and Hani Tamim; Data Acquisition: Moustafa Al Hariri; Writing - original draft preparation: Moustafa Al Hariri, Sally Al Hassan, Malak Khalifeh, Hani Tamim, Imad El Majzoub, and Tharwat El Zahran; Writing - review and editing: Moustafa Al Hariri, Sally Al Hassan, Malak Khalifeh, Hani Tamim, Imad El Majzoub, and Tharwat El Zahran; Funding acquisition: Not applicable; Resources: Moustafa Al Hariri and Malak Khalifeh; Supervision: Tharwat El Zahran.

**Acknowledgements:** The APC of this article was covered by the Qatar National Library.

**Presentations:** The results of this study were presentation at the XII^th^ Mediterranean Emergency Medicine Congress (MEMC) held between 7 and 10 September 2023 in Rhodes, Greece.

**Figures legend:**

**Figure 1: Association of CI-AKI and Baseline eGFR.** The participants were stratified according to the grade of AKI depending on their baseline eGFR level: G1 with eGFR ≥ 90, G2 between 89 and 60, G3 between 59 and 30, G4 between 29 and 15, and G5 <15. Blue bars represent the percentage of the participants who did not show AKI and Red bars represent the percentage of the participants who showed AKI.
