# Supplementary material for: Factors associated with contrast-associated acute kidney injury in an emergency department: A cohort study in Lebanon
Source: PLoS One. 2025 Mar 13;20(3):e0316604. doi: 10.1371/journal.pone.0316604 (PMC11906086; doi:10.1371/journal.pone.0316604)
Supplement: Attachment — Submitted filename: Point by point response Nephrotox.docx [file pone.0316604.s003.docx]

*Dr. Ogochukwu Chinedum Okoye*

*Academic Editor*

*PLoS One*	August 25, 2024

**Dear Dr. Ogochukwu Chinedum Okoye,**

**On behalf of the authors of our submitted manuscript “Contrast-Associated Acute Kidney Injury: an Observational, Cohort Study in an Emergency Department of a Tertiary Care Center” with submission ID PONE-D-24-11651, I would like to thank you and the reviewers for the time to review and suggest improvements for our manuscript. I am submitting herein the point-by-point response addressing the comments and suggestions of the editor(s) and the reviewers.**

Dear Dr. El Zahran,

Thank you for submitting your manuscript to PLOS ONE. After careful consideration, we feel that it has merit but does not fully meet PLOS ONE’s publication criteria as it currently stands. Therefore, we invite you to submit a revised version of the manuscript that addresses the points raised during the review process.

The term contrast-associated AKI (CA-AKI) is more appropriate and should be used throughout the report. The logistic regression tables should be presented in one table. Clearly state in your statistical plan 1) how predictor variables were selected for the logistic regression analysis 2) How were the confounding variables identified?. Although it is important to include medications and clinical parameters as predictor variables, the authors should check for **multicollinearity** and address them as appropriate. For any two variables with r > 0.8, only one should be included in the table.  In addition, address all concerns raised by both reviewers.

**Response: We thank the reviewer for summarizing the reviewers’ points. We have amended the use of contrast induced AKI into Contrast Associated AKI (CA-AKI) on multiple occasions in the manuscript.**

**Logistic regression analysis is presented in table 3 of the manuscript. We have reported the**

**As for the variables that were selected to be tested in the logistic models, we have amended our method section to clarify the selection process and we added to the footnote of Table 3 the variables that were accounted for upon generating the logistic regression model.**

**As for the collinearity of the variables included in the logistic regression model, we have performed Variance Inflation Factor (VIF) values. In the below table, we present the VIF values of the variables, which were all below 5, indicating a low collinearity between the variables.**

| **Variable** | **VIF value** |
| --- | --- |
| **Age (≥ 60 years)** | **1.086826** |
| **SBP (≥ 140)** | **1.053905** |
| **RR (≥ 22)** | **1.067888** |
| **Chronic Kidney Disease** | **1.020159** |
| **Loop Diuretic** | **1.142967** |
| **Beta-Lactams** | **1.120602** |
| **Allopurinol** | **1.018106** |
| **Vancomycin** | **1.130351** |

3. In the online submission form, you indicated that "The datasets generated during and/or analysed during the current study are available from the corresponding author on reasonable request."
 
All PLOS journals now require all data underlying the findings described in their manuscript to be freely available to other researchers, either 1. In a public repository, 2. Within the manuscript itself, or 3. Uploaded as supplementary information.
This policy applies to all data except where public deposition would breach compliance with the protocol approved by your research ethics board. If your data cannot be made publicly available for ethical or legal reasons (e.g., public availability would compromise patient privacy), please explain your reasons on resubmission and your exemption request will be escalated for approval.

**Response: We thank the editor and the reviewers for this suggestion. We have deposited our dataset in Qatar University data repository, which is accessible through the following link: <https://qspace.qu.edu.qa/handle/10576/57874>**

**The data sharing statement in the manuscript was amended accordingly.**

Reviewers' comments:

Reviewer's Responses to Questions

**Comments to the Author**

1. Is the manuscript technically sound, and do the data support the conclusions?

The manuscript must describe a technically sound piece of scientific research with data that supports the conclusions. Experiments must have been conducted rigorously, with appropriate controls, replication, and sample sizes. The conclusions must be drawn appropriately based on the data presented.

Reviewer #1: Partly

Reviewer #2: Yes

2. Has the statistical analysis been performed appropriately and rigorously?

Reviewer #1: No

Reviewer #2: Yes

3. Have the authors made all data underlying the findings in their manuscript fully available?

The [PLOS Data policy](http://secure-web.cisco.com/1Om6nXIYWL_0ChQ0qxAJubhN2iLhn_8kbRk2GSMtjrmgjh55b3nhmlYroc1FFNPHQ3QdpqzV2NYcWWRNCG0q1B61TAi5r0m2D0eH9-FjhbU2d4URkCYL0mWaIHmL60Hlwky1Nul_85SgviKe7fPKTVdzuVtqVsmOjqWFzR-lU_CLWTs57A1bplCfHeMVcuvP-6Xkd4WIlX2WwhLlmTgSjzp-vGgPyLzHHqWCLELAR9W--vAPZ7nX8m2Ky76u2-NB5hW_6Q7Z1C6IYYlnRwecZw1ets32qC-UbJy_kDy2mUSh4f02PFDuArcKJDc7UpoEprZn-vCzWHzMfkIBK3AHcAFMMLz_eDXJAUZitEK5F0JAPSFcK_ysGnsj62aIZEgPEwbQZyt3DzahQ6NNrDB6yXA/http%3A%2F%2Fwww.plosone.org%2Fstatic%2Fpolicies.action%23sharing) requires authors to make all data underlying the findings described in their manuscript fully available without restriction, with rare exception (please refer to the Data Availability Statement in the manuscript PDF file). The data should be provided as part of the manuscript or its supporting information, or deposited to a public repository. For example, in addition to summary statistics, the data points behind means, medians and variance measures should be available. If there are restrictions on publicly sharing data—e.g. participant privacy or use of data from a third party—those must be specified.

Reviewer #1: No

Reviewer #2: No

4. Is the manuscript presented in an intelligible fashion and written in standard English?

PLOS ONE does not copyedit accepted manuscripts, so the language in submitted articles must be clear, correct, and unambiguous. Any typographical or grammatical errors should be corrected at revision, so please note any specific errors here.

Reviewer #1: Yes

Reviewer #2: Yes

5. Review Comments to the Author

Please use the space provided to explain your answers to the questions above. You may also include additional comments for the author, including concerns about dual publication, research ethics, or publication ethics. (Please upload your review as an attachment if it exceeds 20,000 characters)

Reviewer #1: Overview: The authors present the results from a single center, retrospective study of 1,832 emergency department patients who underwent CT with contrast between November 2018 and December 2019. They report a prevalence of contrast-induced acute kidney injury (CI-AKI) of 10.9%, and found that BP≥140mmHg, RR≥22, existing history of CKD, and use of several medications (loop diuretics, beta-lactams, allopurinol) were associated with CI-AKI in a logistic regression analysis.

Major Comments Overall:
- One major issue that must be addressed is that the authors do not differentiate between contrast-associated AKI (CA-AKI) and CI-AKI. CA-AKI, implies a correlative diagnosis of AKI, whereas CI-AKI implies a causative diagnosis. In 2020, the American College of Radiology and the National Kidney Foundation released joint guidelines, published simultaneously in Radiology and Kidney Med (Davenport et al., 2020) that distinguishes between the two. This study must be cited in this paper. The authors methodologic approach does not provide a control group for analysis, therefore no causative link can be drawn between the AKI these patients developed and the contrast they received. Thus, it is crucial that “CI-AKI” be replaced by “CA-AKI” throughout the manuscript. Additionally, this difference should be briefly discussed in the introduction and the discussion, as it helps interpret the authors’ findings.

**Response: We appreciate the reviewer’s insightful comment. We have addressed this issue by making the following changes to the manuscript:**

**We have replaced "CI-AKI" with "CA-AKI" throughout the manuscript to reflect the appropriate terminology as per the guidelines from the American College of Radiology and the National Kidney Foundation (Davenport et al., 2020).**

**We have added the following statement to the Background section to differentiate between CA-AKI and CI-AKI:**

***"*** ***In 2020, the American College of Radiology and the National Kidney Foundation released joint guidelines distinguishing between CA-AKI and contrast-induced acute kidney injury (CI-AKI). CA-AKI refers to AKI that occurs in temporal association with contrast media administration but does not imply causation, whereas CI-AKI implies a direct causal relationship between contrast media and AKI (Davenport et al., 2020)."***

**We have also added the following sentences at the end of the Introduction section to briefly discuss the difference between CA-AKI and CI-AKI:**

***"It is important to differentiate between CA-AKI and CI-AKI. CA-AKI is a correlative diagnosis indicating that AKI occurred after contrast administration, while CI-AKI implies a causative diagnosis. This distinction is crucial as our study design does not allow for a causal inference. Therefore, we will use the term CA-AKI in this manuscript (Davenport et al., 2020)."***

**We have added the following paragraph to the Discussion section to further elaborate on the difference between CA-AKI and CI-AKI and its relevance to our findings:**

***"Our study identified several factors associated with the development of CA-AKI in patients receiving contrast media. It is essential to note that CA-AKI refers to AKI occurring in temporal association with contrast media administration without implying causation, whereas CI-AKI suggests a direct causal relationship. Given the retrospective design of our study and the absence of a control group, we cannot establish a causative link between contrast media and AKI. This aligns with the guidelines from the American College of Radiology and the National Kidney Foundation, which recommend using the term CA-AKI to avoid overstating the risk of contrast media (Davenport et al., 2023, Abbasi et al., 2022, & Weinreb et al. 2021)."***

**References:**

- **Davenport, M. S., et al. (2020). Use of Intravenous Iodinated Contrast Media in Patients with Kidney Disease: Consensus Statements from the American College of Radiology and the National Kidney Foundation. Radiology and Kidney Medicine.**
- **Davenport MS, Perazella MA, Nallamothu BK. Contrast-Induced Acute Kidney Injury and Cardiovascular Imaging: Danger or Distraction? Circulation. 2023;147(11):847-9. doi: doi:10.1161/CIRCULATIONAHA.122.062783.**
- **Abbasi N, Glazer DI, Saini S, Sharma A, Khorasani R. Utility of Patient-Reported Risk Factors for Identifying Advanced Chronic Kidney Disease Before Outpatient CT: Comparison With Recent ACR/NKF Consensus Criteria. American Journal of Roentgenology. 2022;219(3):462-70. doi: 10.2214/ajr.22.27494. PubMed PMID: 35383485.**
- **Weinreb JC, Rodby RA, Yee J, Wang CL, Fine D, McDonald RJ, et al. Use of Intravenous Gadolinium-based Contrast Media in Patients with Kidney Disease: Consensus Statements from the American College of Radiology and the National Kidney Foundation. Radiology. 2021;298(1):28-35. doi: 10.1148/radiol.2020202903. PubMed PMID: 33170103.**

Abstract:
- Replace “CI-AKI” with “CA-AKI” throughout abstract

**Response: We thank the reviewer for this suggestion. We replaced CI-AKI throughout the manuscript with CA-AKI.**

- In the conclusions section, you mention “age” as a factor to consider, but this was not significantly associated with CA-AKI in your model. Please remove.

**Response: We thank the reviewer for this suggestion. However, we respectfully disagree. Our logistic regression model, presented in Table 3, indicates that age (specifically, being 60 years or older) is significantly associated with a higher likelihood of developing CA-AKI. The adjusted odds ratio (aOR) for age being 60 years or older is 1.547, with a 95% confidence interval of 1.091 to 2.195 and a p-value of 0.014. Therefore, age should be included in the conclusions as a factor associated with CA-AKI.**

Introduction:
- Please provide citations throughout for each sentence that cites data or concepts from other studies

**Response: We thank the reviewer for this suggestion. We added citation indications for the data and concepts included in the background section. The added citations are:**

**3.	Andreucci M. Iodinated Contrast-Induced Acute Kidney Injury. Pathophysiology and Prevention. Giornale di Tecniche Nefrologiche e Dialitiche. 2017;29(1):11-9. doi: 10.5301/gtnd.2017.16584.**

**4.	Ye J, Liu C, Deng Z, Zhu Y, Zhang S. Risk factors associated with contrast-associated acute kidney injury in ST-segment elevation myocardial infarction patients: a systematic review and meta-analysis. BMJ Open. 2023;13(6):e070561. doi: 10.1136/bmjopen-2022-070561.**

**5.	Pistolesi V, Regolisti G, Morabito S, Gandolfini I, Corrado S, Piotti G, et al. Contrast medium induced acute kidney injury: a narrative review. Journal of Nephrology. 2018;31(6):797-812. doi: 10.1007/s40620-018-0498-y.**

**7.	Maioli M, Toso A, Leoncini M, Musilli N, Grippo G, Ronco C, et al. Bioimpedance-Guided Hydration for the Prevention of Contrast-Induced Kidney Injury: The HYDRA Study. Journal of the American College of Cardiology. 2018;71(25):2880-9. doi: https://doi.org/10.1016/j.jacc.2018.04.022.**

Methods:
- The study aim should be moved to the last sentence of the introduction

**Response: We thank the reviewer for the suggestion. We amended the manuscript write-up according to the suggestion.**

- It is unclear what variables you adjusted for in your logistic regression model: please clarify which variables either in the methods, or in table 2 where you report regression outputs

**Response: we thank the reviewer for this question. We have clarified the variables adjusted for in our logistic regression model as follows:**

**We have presented the bivariate analysis (chi-square or independent t-test) of our variables in Tables 1 and 2. We also added the False Discovery Rate (FDR) value of the results to account for multiple comparisons.**

**We assessed and presented the unadjusted odds ratio (OR) of each variable to assist in selecting the variables for the logistic regression model. We relied on the unadjusted OR confidence interval rather than just the p-value or q-value from the bivariate analysis.**

**The results of the logistic regression are presented in Table 3. The variables selected for the logistic regression model are listed as a footnote underneath Table 3.**

**The Methods section describing the selection of variables for the logistic regression reads as follows:**

***“Logistic regression was then done to adjust confounding variables and test the association between CA-AKI and other variables. Variables that showed a significant confidence interval of the unadjusted OR and clinically relevant were selected to build the logistic regression model.”***

Results:
- For Table 2, is this a multiple logistic regression model or are these bivariate p values? If it was a multiple logistic regression model, it would be helpful to show the bivariate analysis to see how ORs changed after being included in the multivariate model

**Response: We thank the reviewer for this important question. Table 2 presents the bivariate p-values from our analysis and not multiple logistic regression models. We added the unadjusted odds ratio (OR) for each of the variables and their 95% confidence intervals (95% CI) to this table to aid in selecting factors for the logistic regression model presented in Table 3.**

**To enhance clarity, we have updated Tables 1 and 2 to clearly indicate that they present bivariate p-values and unadjusted ORs. Additionally, we have included footnotes in Tables 1 and 2 to clarify that the ORs and the 95% CIs presented in these tables are generated from unadjusted logistic regression.**

**The modified footnotes for tables 1 and 2 read as follows:**

***“Data are presented as numbers with percentages.***

***The p-value for the difference between AKI vs. no AKI is calculated by chi-square, Fisher´s exact test, or T-test where appropriate.***

***The q-value is the False Discovery Rate (FDR) significance accounting for multiple comparisons.***

***The unadjusted Odd Ratio (OR) for each variable was calculated without accounting for any other factor. 95% CI is the confidence interval of the OR for each variable.”***

- For table 2, it would be helpful to know the row percentages: For example: how many patients with qSOFA 0, 1 or 2 had AKI? How many patients on loop diuretics had AKI compared to those not on loop diuretics? These are the proportions that matter so we can understand how prevalence of AKI differs by each characteristic

**Response: We thank the reviewer for this suggestion. We understand the importance of providing clear and comprehensive data to enhance the understanding of our findings. However, we believe that reporting column percentages is sufficient and more intuitive for interpreting the data in the context of our study. Column percentages allow readers to easily compare the prevalence of AKI across different categories of each variable.**

**Additionally, since we have included the total number of participants for each variable, the row percentages can be easily deduced if needed. For example, the total number of patients with qSOFA scores of 0, 1, and 2, as well as the number of patients on loop diuretics, are clearly presented. Readers can calculate the row percentages by dividing the number of patients with AKI by the total number of patients in each category.**

**We believe that maintaining the column percentages helps to avoid potential confusion and keeps the tables concise and readable. However, we are open to further discussion if the reviewer feels strongly about including row percentages.**

**Therefore, we have retained the column percentages in Table 2 to provide a clear and intuitive presentation of the data. Additionally, We have ensured that the total number of participants for each variable is clearly presented, allowing readers to easily deduce row percentages if needed.**

**We hope this explanation addresses the reviewer's concern and maintains the clarity and readability of our tables.**


Discussion:
- It is interesting that BP and RR are associated with CA-AKI; but how do you suggest interpreting this? It is very likely that elevated BP is simply a marker of undiagnosed hypertension, which itself is predictive of AKI during any admission (Jiang 2021, BMC Nephrology). Similarly, elevated RR may be a marker of acuity of illness; this elevated acuity of illness likely predisposes an increased propensity to AKI regardless of contrast administration. For example, conditions such as volume overload and sepsis would increase the RR and both are known to contribute to AKI. Indeed, it was unsurprising that AKI was more common in the qSOFA 1 group. These diagnoses also increase the likelihood that someone might get a contrast study to evaluate for infection or pulmonary embolism, for example. This must be discussed.

**Response: we thank the reviewer for these suggestions. We have included our discussion parts related to the hypertension, respiratory rate, and sepsis.**

- Must add lack of control group to the limitations part of discussion

**Response: We thank the reviewer for this point. We added to the limitation section the lack of a control group.**

Reviewer #2: We would like to thanks to the authors for giving me the opportunity to review the manuscript entitled "Factors Associated with Contrast-Induced Acute Kidney Injury in a Tertiary Care Center Emergency Department: an Observational Cohort Study".
The manuscript is very interesting, but needs a few changes to improve it.
Introduction
Why have the authors placed the objective of the study in the method section? We believe that the objective of the study should be placed at the end of the context.

**Response: We thank the reviewer for this suggestion. We moved the objective to the end of the introduction section.**

Method
This study was approved by the IRB,
Authors should explain the abbreviation at the first appearance in the text.

**Response: We thank the reviewer for this suggestion. We have reviewed the manuscript's text and defined the abbreviations on their first appearance.**

Study approval may be presented in a different section as an Ethics Statement.

**Response: We thank the reviewer. We have the ethical statement included in the manuscript in the declaration section after references.**

The authors must correctly state the inclusion criteria: did the authors include all patients? What is the age criterion for inclusion?

**Response: We thank the reviewer for the question. We included the records of the adult patients who underwent contract induced computer topography during their ED visit during the study period. We have reviewed the “Population” section of the methods.**

The authors report that patients receive a mean contrast dose of 2ml/kg. As this is an average, could the authors add the standard deviation?

**Response: We thank the reviewer for this question. The contrast administration, specifically using Omnipaque 350, is part of the standard medical procedure carried out by healthcare professionals at our institution and is not directly related to the study's data collection process. Typically, patients receive 1 ml/kg for routine imaging and 1.5 ml/kg for angiographic procedures if their creatinine levels are normal.**

**As researchers, we do not have access to the exact dosage used for each patient, which limits our ability to calculate the standard deviation of the contrast dose. The mean dose of 2 ml/kg reported in the study is an average estimation based on institutional protocols rather than patient-specific data. Given these constraints, we are unable to provide the standard deviation for the contrast dose.**

**We hope this explanation clarifies the context of the contrast dose information and the limitations in reporting the standard deviation.**

**We have updated the population section of the study to include the details of the contrast solution dosage.**

The author should specify the type of iodinated contrast medium used: iso or low-osmolarity?

**Response: We thank the reviewer for the question. The contrast medium administered to patients was Omnipaque 350, which is classified as a low-osmolar contrast medium.**

**We have included these details in the population section of the manuscript.**

In the methods section
Outcome measures: this title would normally correspond to data collection.
home medication, vitals, ED management (medications and IV administration), home medications,
In this sentence, home medication is repeated

**Response: We thank the reviewer for this request. We have modified the title of this section to “Data collection,” and we have removed one of the repeated “home medications”.**

laboratory workup
The authors should specify which laboratory data were collected and reported in Table 1, where the general characteristics of the study are presented according to AKI status.

**Response: We thank the reviewer for this question. Creatinine was the laboratory workup assessed in the study. An indication of this was added to the method section.**

Could the authors provide information on the stage of AKI and incidence of hemodialysis in these AKI-IC cases?

**Response: We appreciate the reviewer's interest in further details regarding the stage of AKI and the incidence of hemodialysis among AKI-IC cases.**

**To address the first part of the question, we have generated Figure 1 that stratifies patients into AKI and non-AKI groups based on their baseline estimated glomerular filtration rate (eGFR) prior to contrast-enhanced computed tomography (CI-CT). This stratification provides insights into the renal function status of patients at baseline. However, it is important to note that this graph does not directly correspond to the specific stages of AKI as defined by KDIGO criteria, which are based on changes in serum creatinine and urine output. The graph provides valuable baseline information but may not fully capture the specific stages of AKI.**

**Regarding the incidence of hemodialysis, we do not have access to the detailed medical records required to determine how many of the CA-AKI cases required hemodialysis. The data for our study was extracted by the Medical Records team at our institution, and we did not review individual patient charts. Therefore, we are unable to provide the incidence of hemodialysis among these cases at this stage.**

Definition of AKI
as an increase in creatinine of 0.3 mg/dl (26 mmol/l) within 48 hours: please correct the unit to 26 µmol/L

**Response: We thank the reviewer for pointing out this typing mistake. We have amended the units in the AKI definition.**

The title definition of AKI should be replaced by an operational definition in which the authors could report the definition of Aki and add the definition of CKD.

**Response: We thank the reviewer for this suggestion. We have modified the section title and added a definition for CKD.**

Statistical analysis

Continuous variables were presented using mean and standard deviation (mean ± SD).
The authors can confirm that all data were normally distributed, as they did not present the results as median (IQR).

**Response: We thank the reviewer. We removed this statement from the method section since all our reported data on the participants were categorical. The newly reported creatinine variable is non-linear. Therefore, the median and interquartile ranges were reported in Table 2. A note was added to the footnote of Table 2 and to the method section.**

Results
In Table 1, what is the difference between antiviral and antiretroviral drugs?

**Response: We thank the reviewer for the question. We apologize for keeping the antiretroviral variable on the table. The antiviral included Acyclovir, Cidofovir, Foscarnet, and Valganciclovir. The Antiretroviral included Indinavir. However, since the latter was observed only once in our study, we will remove the variable from the dataset and Table 2 to streamline the analysis and focus on the relevant medications. Additionally, we have removed the following medications from Table 2 for the low number of observations: Dapsone, Rifampicin, and Tetracycline.**

In Table 1, the authors should compare baseline creatinine values between the AKI and non-AKI groups.

**Response: We thank the reviewer for this suggestion. The baseline creatinine levels were added to Table 2. We revised the FDR (q-values) for all covariates and updated the ones that have been modified by the added and removed variables. A description of the statistical analysis test used for analyzing the baseline creatinine level was added to the method section.**

Patients who received lithium (30.5% vs. 22%, p=0.008): these results do not correspond to those reported in Table 1.

**Response: We thank the reviewer for pointing out this mistake. We have reviewed the result section of the manuscript and aligned it with what is reported in Table 2.**

Patients with high blood pressure (≥140 mmHg): this result is not consistent with that reported in Table 3.

**Response: we thank the reviewer for this question. We reported the rounded up value of the aOR and the CI in the narrative section of the results related to table 3 in the manuscript. We have reviewed the numbers reported in Table 3 and rounded them up similarly to the narrative section to avoid any inconsistency and confusion for the readers.**

Why were many factors significant in the univariate logistic regression not presented in Table 3 in the multivariate logistic regression?

**Response: We thank the reviewer for this question. We actually included all statistically significant variables reported in Tables 1 and 2 into the logistic regression model presented in Table 3.**

Please write ˂ 0.001 for the p-value of 0.000.

**Response: We thank the reviewer for this suggestion. All p values were revised and adjusted according to this suggestion.**

Limitation
Please correct the KDIGO definition of acute renal failure. For KDIGO

Translated with [www.DeepL.com/Translator](http://www.DeepL.com/Translator) (free version)

6. PLOS authors have the option to publish the peer review history of their article ([what does this mean?](https://secure-web.cisco.com/1zjfSg3ZI_XVy-QfkAhSHsF1roTLLIreI4JJjXjai_GysfjhROIyh-WCcEu11WKsgLQtaDELZuKH5qz9oTvFfKNp22gAa0L_iOwO_-Rdc7DxlW6Dx5s722X0lXJY6wnbxEtFyuCmaqkyBPMItJrpY0WTpXl7N7QdmaC9cWDCkQwhDFOB3j5FwalJF-a7t6FpVK05b9pff3sbTSE5aISDqHfGMGwaazJKTG7dzHGqF2Gv_omdjersK0Z7iRD41HEk5P5VapAckOPBNZMt1P66yPDyFhN249RsHXNDS07NqZ-y_3Mmeai5wucfBHBrfrNZci9kRGi4odwXGi-DR_2X_tdvracBHQGupGEz9x4oy09m4flw35xy52xyMlkS2u0dYgXAhBcOVhf6gyp3AgkOuXQ/https%3A%2F%2Fjournals.plos.org%2Fplosone%2Fs%2Feditorial-and-peer-review-process%23loc-peer-review-history)). If published, this will include your full peer review and any attached files.


If you choose “no”, your identity will remain anonymous but your review may still be made public.


**Do you want your identity to be public for this peer review?** For information about this choice, including consent withdrawal, please see our [Privacy Policy](https://secure-web.cisco.com/1sCd2ItrC4hPe3px49g7WHc-r5J6BWqwVmc_NIoyxeVXJaGq4t-jL0ugBWcJ0QE9ToFG9kUlm_kJnJCheCteYA5Rr9HGUj4KqhCu0IXofCiuacRfMJjUSDI0UtuIUK4CMPLgl5j1jvXND2to7-hZBaL6DHMZRkhrffdeY6b8nKQZOOz3JrXXUHGmC75kQWqKPoKu1vvaDBrrc_qvtx6mKKgX8yg1BfVUX2PeiCHq3HA_jSFqGCvcekHw_oQm9gJ33T07gcYjsXuU2cXJYlWbaAMpTa_JW6lu4LHqiTUyYlnKukvXqY1iwfAmsfaPQp9FJ9rypjuVyZLBAq5dh0GlDrgxMizJCEdm0O3rCr8-KJK2eUMzsdSKFwmdxkJpvCSm0uzqiO4pLWY1uug1LhQmdbg/https%3A%2F%2Fwww.plos.org%2Fprivacy-policy).

Reviewer #1: No

Reviewer #2: **Yes:** yannick nlandu

[NOTE: If reviewer comments were submitted as an attachment file, they will be attached to this email and accessible via the submission site. Please log into your account, locate the manuscript record, and check for the action link "View Attachments". If this link does not appear, there are no attachment files.]

While revising your submission, please upload your figure files to the Preflight Analysis and Conversion Engine (PACE) digital diagnostic tool, [https://pacev2.apexcovantage.com/](https://secure-web.cisco.com/1OorovvV7HiwnoVK7yLQp1YEV2Gh1ak4kHihVH1epvZi2GVS8KbERRv9auiFauTO9awPTBGz-asPf8mTEs5_l6eaw2_HNq2MqZ_qb2OPxBITkC7XgmSbLGMW6TAfhhy6N-vluzu0mnoagWE3E15QAhwKuOJaxt15ntzcD8vym8VKWrYV10OwZWHC2q0A3PQvzm97ROqnssFMQEsoKKLpZ3BVpGtWJkfyeuw6e_RpnTP-R6qm3Q8cAKtmI3mzeDKnMCy1-9ShfvueX3iGL39t3LJuZaBuIexxFuAJBPqY5HrZEh_TAz7o0ADsGm7Ap1KoQDwAPZPnUfS2jCJ9S8Zzfcxo0Dw_eW1FYsKEJyy1IgDU5H0NabWIjJozIUO2b2QbiQmMFHx-1NoN2TM9_Ucb73w/https%3A%2F%2Fpacev2.apexcovantage.com%2F). PACE helps ensure that figures meet PLOS requirements. To use PACE, you must first register as a user. Registration is free. Then, login and navigate to the UPLOAD tab, where you will find detailed instructions on how to use the tool. If you encounter any issues or have any questions when using PACE, please email PLOS at [figures@plos.org](mailto:figures@plos.org). Please note that Supporting Information files do not need this step.
