# Supplementary material for: Factors associated with contrast-associated acute kidney injury in an emergency department: A cohort study in Lebanon
Source: PLoS One. 2025 Mar 13;20(3):e0316604. doi: 10.1371/journal.pone.0316604 (PMC11906086; doi:10.1371/journal.pone.0316604)
Supplement: Attachment — Submitted filename: Reviewer responses-2.docx [file pone.0316604.s004.docx]

Point-by-point Response	Date: December10, 2024

We thank the editor and the reviewers for sharing their suggestions to improve our manuscript “Factors Associated with Contrast-Associated Acute Kidney Injury in a Tertiary Care Center Emergency Department: an Observational Cohort Study” with submission number “PONE-D-24-16070R1.”

We would like to indicate that the title of the manuscript was modified according to the suggestion on the provided manuscript word document file to the following:

“Factors Associated with Contrast-Associated Acute Kidney Injury in an Emergency Department: A Cohort Study in Lebanon.”

We have reviewed all the comments added to the document and implemented the needed changes. All changes were tracked on the file. Additionally, please find below our point-by-point response to the points raised by the reviewers.

**Reviewers' comments the Author:**

Reviewer #2: We thank the editor for the opportunity to review the revised manuscript.
We feel that the manuscript is improved, but requires some minor change
Introduction
This study aims to assess the factors associated with the development of CA-AKI in patients who received computed tomography (CT) scans with contrast during their presentation to an academic Emergency Department in a tertiary care center. It is essential to differentiate between CA-AKI and CI-AKI. CA-AKI is a correlative diagnosis indicating that AKI occurred after contrast administration, while CI-AKI implies a causative diagnosis. This distinction is crucial as our study design does not allow for a causal inference. Therefore, we will use the term CAAKI in this manuscript [2].
We believe that the second sentence of this paragraph is repetitive and should be deleted. The authors have already correctly distinguished between CA-AKI and CI-AKI in the second paragraph of their introduction.

**Response:** We thank the reviewer for the suggestion. We removed the repetitive statement.

Methods
Study participants received an average of 2ml1ml/Kg for routine CT and 1.5ml/Kg for angio-CT of low-osmolar contrast medium (Omnipaque 350) contrast solution
In front of 1ml/kg please delete '2ml

**Response:** We thank the reviewer for the suggestion. The edits are implemented in the method section.

Patients’ details were prepared as reports by the EPIC team
Please define the term “EPIC”

**Response:** We thank the reviewer for the suggestion. EPIC is the name of the Electronic Health record used in our institution. Therefore, it is defined as such is the method section “Electronic Health Record (EPIC) team .”

Results
Discussion
We also think it would be interesting for the authors of this interesting article to comment on the incidence of CA-AKI reported in their study. In the context where the authors report an association between high respiratory rate and CA-AKI, the higher incidence of CA-AKI in COVID-19 patients (26.1%) (mainly respiratory pathology) could be a further argument in favour of the association between high respiratory rate and CA-AKI. Nlandu Y, Makulo JR, Essig M, Sumaili E, Lumaka A, Engole Y, Mboliasa MF, Mokoli V, Tshiswaka T, Nkodila A, Bukabau J, Longo A, Kajingulu F, Zinga C, Nseka N. Factors associated with acute kidney injury (AKI) and mortality in COVID-19 patients in a Sub-Saharan African intensive care unit: a single-center prospective study. Ren Fail. 2023;45(2):2263583. doi: 10.1080/0886022X.2023.2263583. Epub 2023 Oct 23. PMID: 37870858; PMCID: PMC11001370.

**Response:** We thank the reviewer for the suggestion. We would like to emphasize that the study was conducted before the COVID period, and no COVID-19 patients were included in the study. Therefore, we will not compare our results to the ones reported in the proposed study.

Limitations

On the other hand, the selection bias in our study was reduced by the fact that around 17% of the study participants exhibited an abnormal (above and lower the normal range) baseline creatinine level. Among these 306 participants, only 42 fulfilled the KODIGO definition of AKI.

Please correct KODIGO to KDIGO

**Response:** We thank the reviewer for the suggestion. We corrected this typing mistake.

Reviewer #3: The Abstract and introduction were well written and captivating with minor errors as highlighted in the attachment .
The aim was clear and succinct
Overall methodology was thorough and aligned well with the study aim. However, it may be clearer to structure the paragraph with bullet points or distinct sections for "Study Design", "Setting", Ethical Approval", for better organization.
The results with tables and illustrations was well captured and discussion was sound as well.
By and large, very fine manuscript.

**Response:** We thank the reviewer for the revision.
